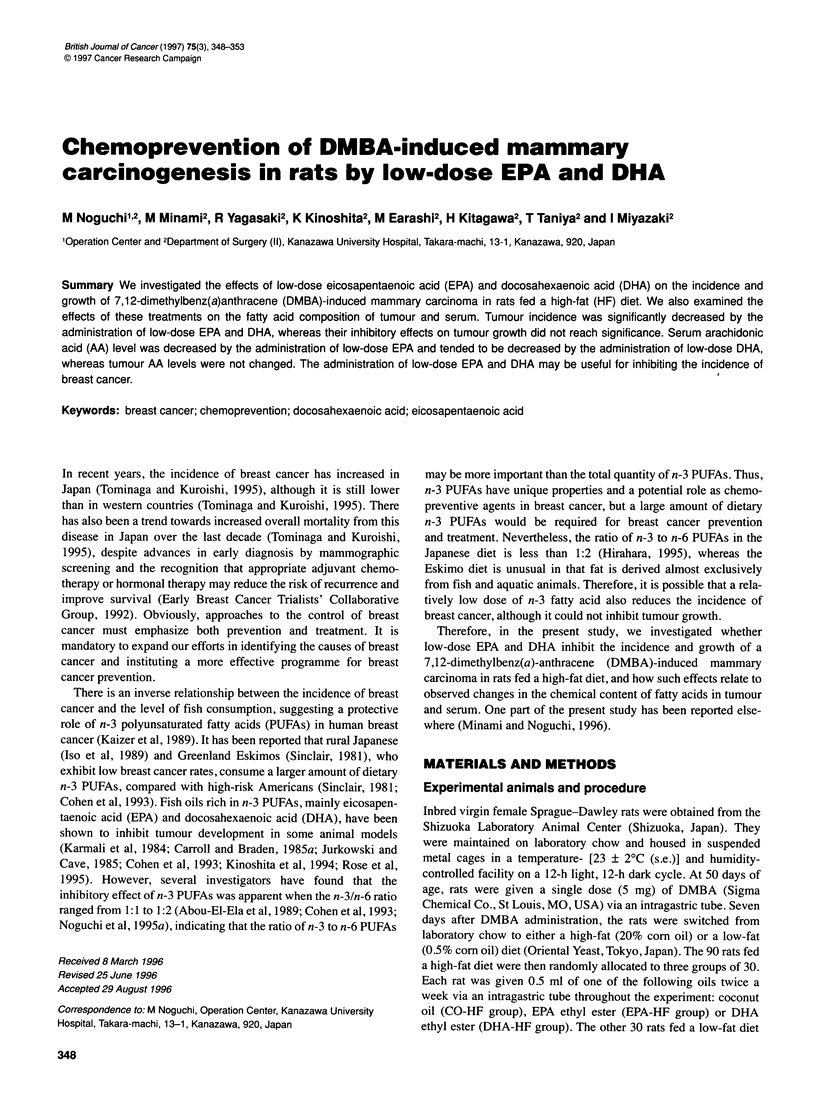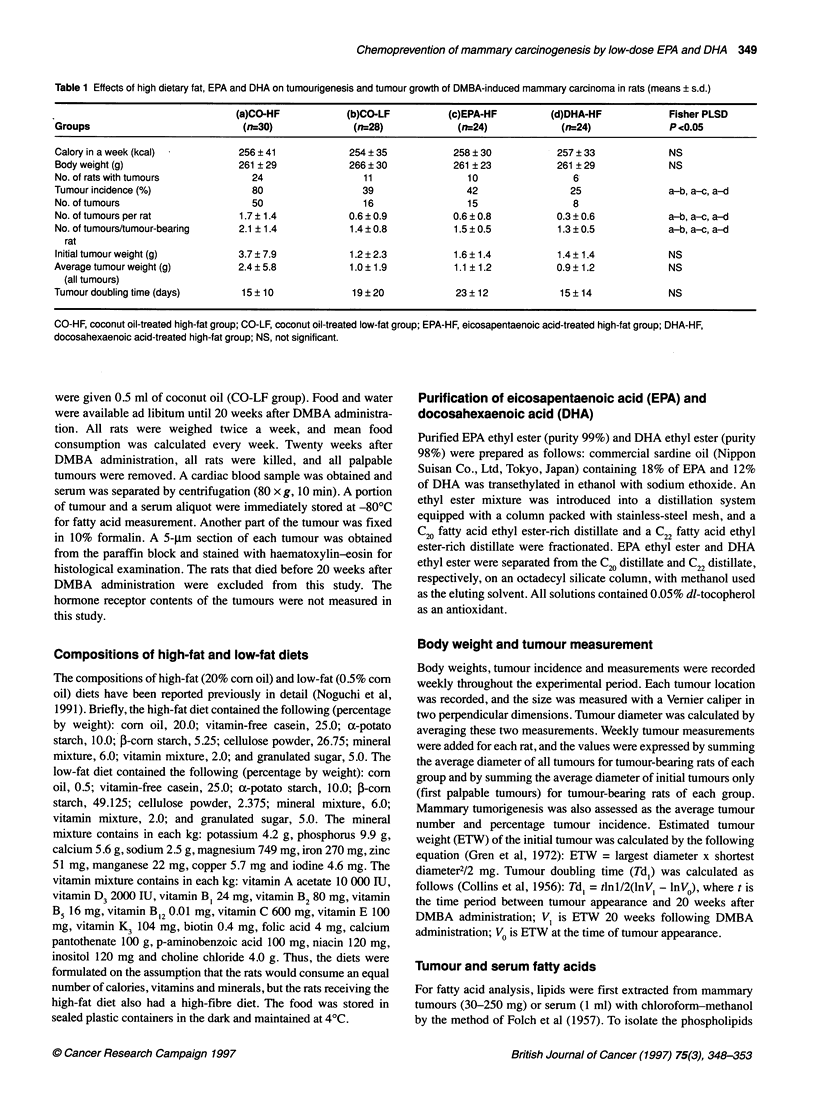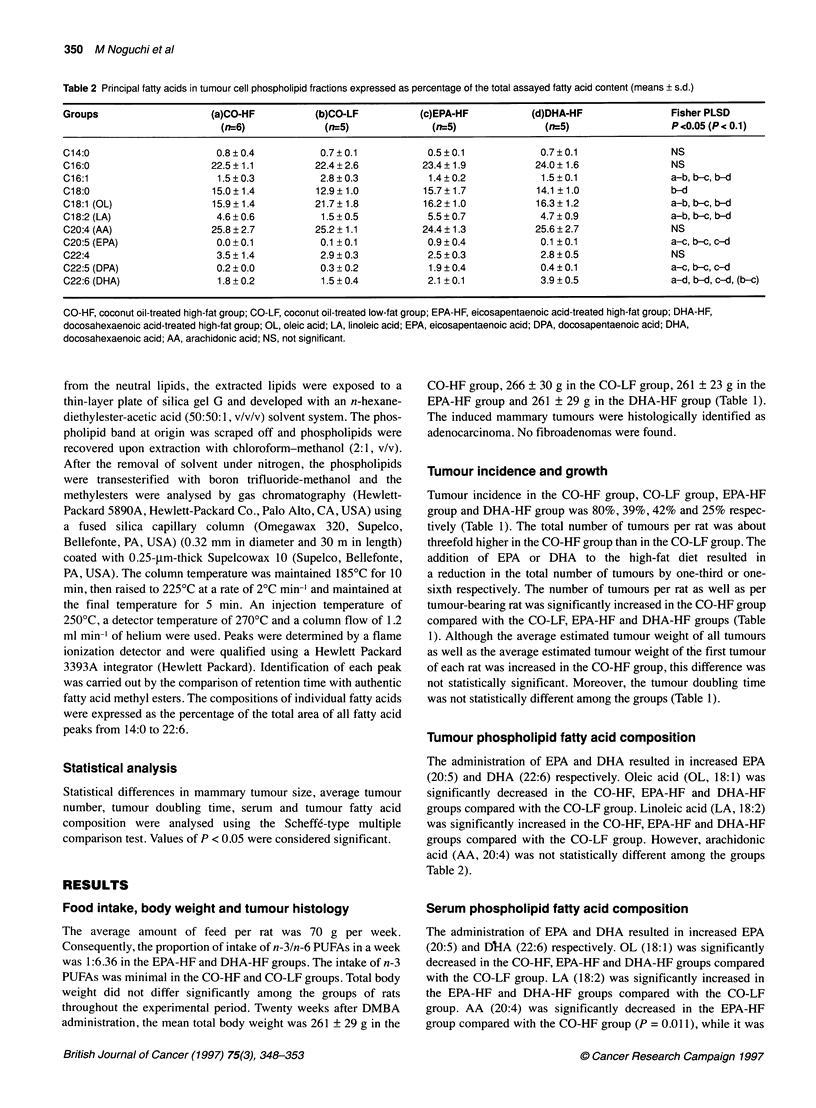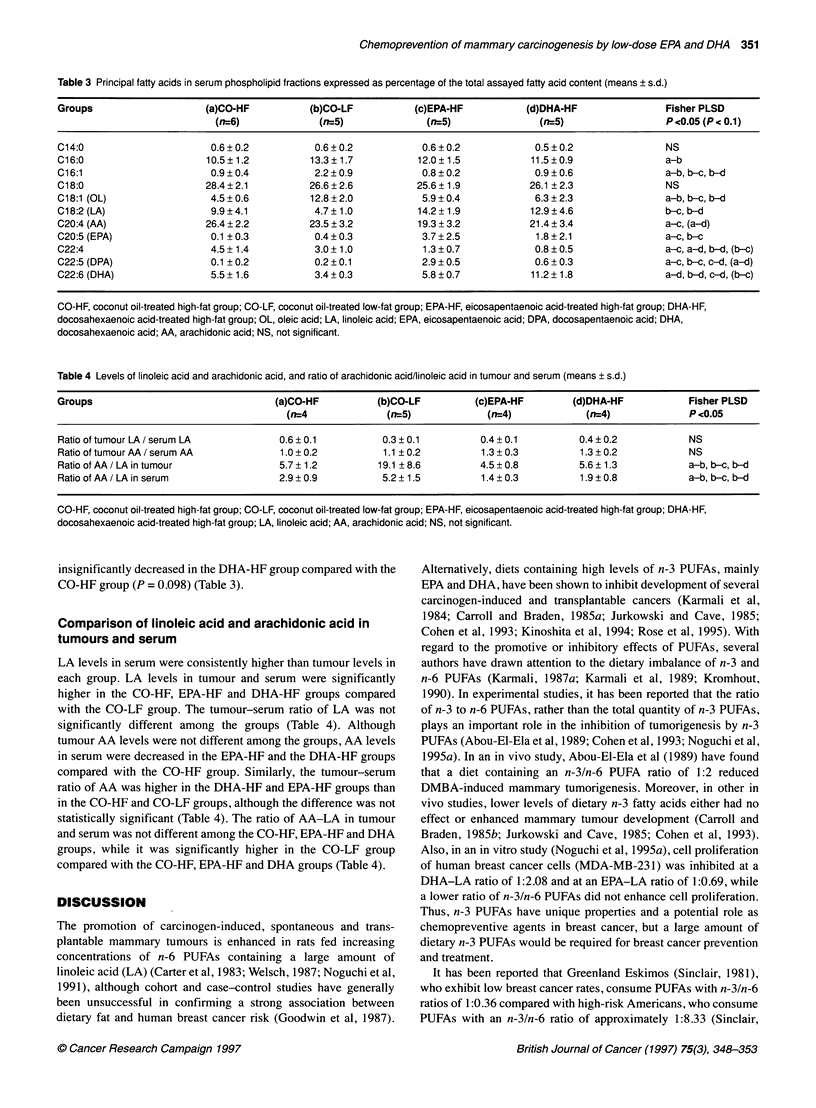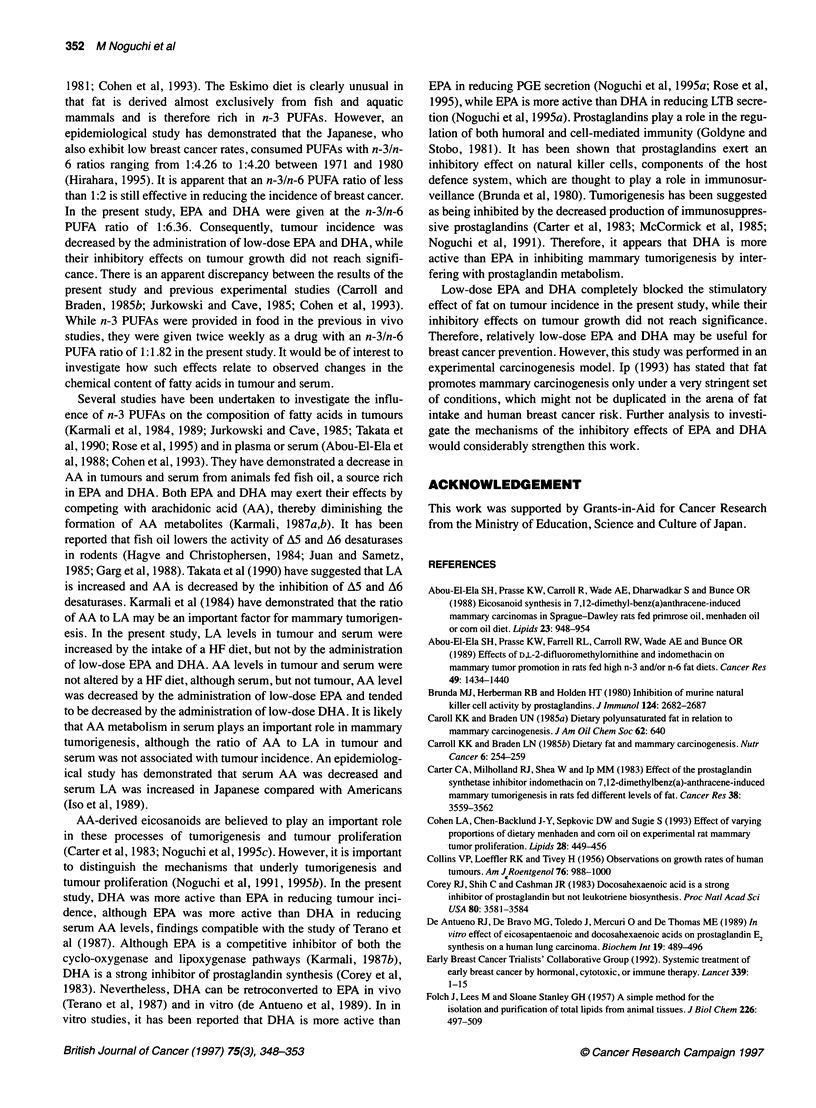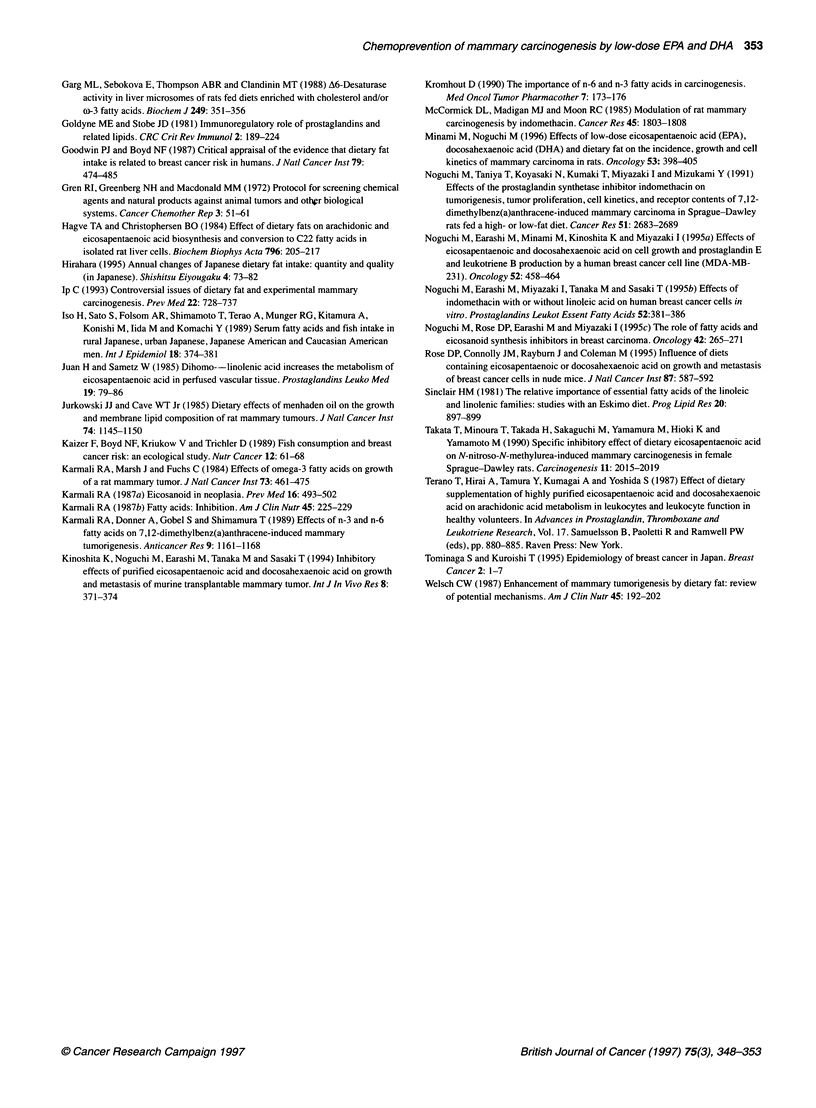# Chemoprevention of DMBA-induced mammary carcinogenesis in rats by low-dose EPA and DHA.

**DOI:** 10.1038/bjc.1997.57

**Published:** 1997

**Authors:** M. Noguchi, M. Minami, R. Yagasaki, K. Kinoshita, M. Earashi, H. Kitagawa, T. Taniya, I. Miyazaki

**Affiliations:** Operation Center, Kanazawa University Hospital, Takara-machi, Japan.

## Abstract

We investigated the effects of low-dose eicosapentaenoic acid (EPA) and docosahexaenoic acid (DHA) on the incidence and growth of 7,12-dimethylbenz(a)anthracene (DMBA)-induced mammary carcinoma in rats fed a high-fat (HF) diet. We also examined the effects of these treatments on the fatty acid composition of tumour and serum. Tumour incidence was significantly decreased by the administration of low-dose EPA and DHA, whereas their inhibitory effects on tumour growth did not reach significance. Serum arachidonic acid (AA) level was decreased by the administration of low-dose EPA and tended to be decreased by the administration of low-dose DHA, whereas tumour AA levels were not changed. The administration of low-dose EPA and DHA may be useful for inhibiting the incidence of breast cancer.


*British Journal of Cancer* (1997) **75**(3), 348–353
© 1997 Cancer Research Campaign

### Chemoprevention of DMBA-induced mammary carcinogenesis in rats by low-dose EPA and DHA

**M Noguchi[1,2], M Minami[2], R Yagasaki[2], K Kinoshita[2], M Earashi[2], H Kitagawa[2], T Taniya[2] and I Miyazaki[2]**

[1]Operation Center and [2]Department of Surgery (II), Kanazawa University Hospital, Takara-machi, 13-1, Kanazawa, 920, Japan

**Summary** We investigated the effects of low-dose eicosapentaenoic acid (EPA) and docosahexaenoic acid (DHA) on the incidence and growth of 7,12-dimethylbenz(*a*)anthracene (DMBA)-induced mammary carcinoma in rats fed a high-fat (HF) diet. We also examined the effects of these treatments on the fatty acid composition of tumour and serum. Tumour incidence was significantly decreased by the administration of low-dose EPA and DHA, whereas their inhibitory effects on tumour growth did not reach significance. Serum arachidonic acid (AA) level was decreased by the administration of low-dose EPA and tended to be decreased by the administration of low-dose DHA, whereas tumour AA levels were not changed. The administration of low-dose EPA and DHA may be useful for inhibiting the incidence of breast cancer.

**Keywords:** breast cancer; chemoprevention; docosahexaenoic acid; eicosapentaenoic acid

In recent years, the incidence of breast cancer has increased in Japan (Tominaga and Kuroishi, 1995), although it is still lower than in western countries (Tominaga and Kuroishi, 1995). There has also been a trend towards increased overall mortality from this disease in Japan over the last decade (Tominaga and Kuroishi, 1995), despite advances in early diagnosis by mammographic screening and the recognition that appropriate adjuvant chemotherapy or hormonal therapy may reduce the risk of recurrence and improve survival (Early Breast Cancer Trialists' Collaborative Group, 1992). Obviously, approaches to the control of breast cancer must emphasize both prevention and treatment. It is mandatory to expand our efforts in identifying the causes of breast cancer and instituting a more effective programme for breast cancer prevention.

There is an inverse relationship between the incidence of breast cancer and the level of fish consumption, suggesting a protective role of *n*-3 polyunsaturated fatty acids (PUFAs) in human breast cancer (Kaizer et al, 1989). It has been reported that rural Japanese (Iso et al, 1989) and Greenland Eskimos (Sinclair, 1981), who exhibit low breast cancer rates, consume a larger amount of dietary *n*-3 PUFAs, compared with high-risk Americans (Sinclair, 1981; Cohen et al, 1993). Fish oils rich in *n*-3 PUFAs, mainly eicosapentaenoic acid (EPA) and docosahexaenoic acid (DHA), have been shown to inhibit tumour development in some animal models (Karmali et al, 1984; Carroll and Braden, 1985*a*; Jurkowski and Cave, 1985; Cohen et al, 1993; Kinoshita et al, 1994; Rose et al, 1995). However, several investigators have found that the inhibitory effect of *n*-3 PUFAs was apparent when the *n*-3/*n*-6 ratio ranged from 1:1 to 1:2 (Abou-El-Ela et al, 1989; Cohen et al, 1993; Noguchi et al, 1995*a*), indicating that the ratio of *n*-3 to *n*-6 PUFAs may be more important than the total quantity of *n*-3 PUFAs. Thus, *n*-3 PUFAs have unique properties and a potential role as chemopreventive agents in breast cancer, but a large amount of dietary *n*-3 PUFAs would be required for breast cancer prevention and treatment. Nevertheless, the ratio of *n*-3 to *n*-6 PUFAs in the Japanese diet is less than 1:2 (Hirahara, 1995), whereas the Eskimo diet is unusual in that fat is derived almost exclusively from fish and aquatic animals. Therefore, it is possible that a relatively low dose of *n*-3 fatty acid also reduces the incidence of breast cancer, although it could not inhibit tumour growth.

Therefore, in the present study, we investigated whether low-dose EPA and DHA inhibit the incidence and growth of a 7,12-dimethylbenz(*a*)-anthracene (DMBA)-induced mammary carcinoma in rats fed a high-fat diet, and how such effects relate to observed changes in the chemical content of fatty acids in tumour and serum. One part of the present study has been reported elsewhere (Minami and Noguchi, 1996).

### MATERIALS AND METHODS

### Experimental animals and procedure

Inbred virgin female Sprague–Dawley rats were obtained from the Shizuoka Laboratory Animal Center (Shizuoka, Japan). They were maintained on laboratory chow and housed in suspended metal cages in a temperature- [23 ± 2°C (s.e.)] and humidity-controlled facility on a 12-h light, 12-h dark cycle. At 50 days of age, rats were given a single dose (5 mg) of DMBA (Sigma Chemical Co., St Louis, MO, USA) via an intragastric tube. Seven days after DMBA administration, the rats were switched from laboratory chow to either a high-fat (20% corn oil) or a low-fat (0.5% corn oil) diet (Oriental Yeast, Tokyo, Japan). The 90 rats fed a high-fat diet were then randomly allocated to three groups of 30. Each rat was given 0.5 ml of one of the following oils twice a week via an intragastric tube throughout the experiment: coconut oil (CO-HF group), EPA ethyl ester (EPA-HF group) or DHA ethyl ester (DHA-HF group). The other 30 rats fed a low-fat diet

*Received 8 March 1996*
*Revised 25 June 1996*
*Accepted 29 August 1996*

*Correspondence to:* M Noguchi, Operation Center, Kanazawa University Hospital, Takara-machi, 13–1, Kanazawa, 920, Japan

**Table 1** Effects of high dietary fat, EPA and DHA on tumourigenesis and tumour growth of DMBA-induced mammary carcinoma in rats (means ± s.d.)

| Groups | (a)CO-HF ($n$=30) | (b)CO-LF ($n$=28) | (c)EPA-HF ($n$=24) | (d)DHA-HF ($n$=24) | Fisher PLSD $P$<0.05 |
|---|---|---|---|---|---|
| Calory in a week (kcal) | 256 ± 41 | 254 ± 35 | 258 ± 30 | 257 ± 33 | NS |
| Body weight (g) | 261 ± 29 | 266 ± 30 | 261 ± 23 | 261 ± 29 | NS |
| No. of rats with tumours | 24 | 11 | 10 | 6 | |
| Tumour incidence (%) | 80 | 39 | 42 | 25 | a–b, a–c, a–d |
| No. of tumours | 50 | 16 | 15 | 8 | |
| No. of tumours per rat | 1.7 ± 1.4 | 0.6 ± 0.9 | 0.6 ± 0.8 | 0.3 ± 0.6 | a–b, a–c, a–d |
| No. of tumours/tumour-bearing rat | 2.1 ± 1.4 | 1.4 ± 0.8 | 1.5 ± 0.5 | 1.3 ± 0.5 | a–b, a–c, a–d |
| Initial tumour weight (g) | 3.7 ± 7.9 | 1.2 ± 2.3 | 1.6 ± 1.4 | 1.4 ± 1.4 | NS |
| Average tumour weight (g) (all tumours) | 2.4 ± 5.8 | 1.0 ± 1.9 | 1.1 ± 1.2 | 0.9 ± 1.2 | NS |
| Tumour doubling time (days) | 15 ± 10 | 19 ± 20 | 23 ± 12 | 15 ± 14 | NS |

CO-HF, coconut oil-treated high-fat group; CO-LF, coconut oil-treated low-fat group; EPA-HF, eicosapentaenoic acid-treated high-fat group; DHA-HF, docosahexaenoic acid-treated high-fat group; NS, not significant.

were given 0.5 ml of coconut oil (CO-LF group). Food and water were available ad libitum until 20 weeks after DMBA administration. All rats were weighed twice a week, and mean food consumption was calculated every week. Twenty weeks after DMBA administration, all rats were killed, and all palpable tumours were removed. A cardiac blood sample was obtained and serum was separated by centrifugation (80 × ***g***, 10 min). A portion of tumour and a serum aliquot were immediately stored at –80°C for fatty acid measurement. Another part of the tumour was fixed in 10% formalin. A 5-μm section of each tumour was obtained from the paraffin block and stained with haematoxylin–eosin for histological examination. The rats that died before 20 weeks after DMBA administration were excluded from this study. The hormone receptor contents of the tumours were not measured in this study.

### Compositions of high-fat and low-fat diets

The compositions of high-fat (20% corn oil) and low-fat (0.5% corn oil) diets have been reported previously in detail (Noguchi et al, 1991). Briefly, the high-fat diet contained the following (percentage by weight): corn oil, 20.0; vitamin-free casein, 25.0; α-potato starch, 10.0; β-corn starch, 5.25; cellulose powder, 26.75; mineral mixture, 6.0; vitamin mixture, 2.0; and granulated sugar, 5.0. The low-fat diet contained the following (percentage by weight): corn oil, 0.5; vitamin-free casein, 25.0; α-potato starch, 10.0; β-corn starch, 49.125; cellulose powder, 2.375; mineral mixture, 6.0; vitamin mixture, 2.0; and granulated sugar, 5.0. The mineral mixture contains in each kg: potassium 4.2 g, phosphorus 9.9 g, calcium 5.6 g, sodium 2.5 g, magnesium 749 mg, iron 270 mg, zinc 51 mg, manganese 22 mg, copper 5.7 mg and iodine 4.6 mg. The vitamin mixture contains in each kg: vitamin A acetate 10 000 IU, vitamin $D_3$ 2000 IU, vitamin $B_1$ 24 mg, vitamin $B_2$ 80 mg, vitamin $B_5$ 16 mg, vitamin $B_{12}$ 0.01 mg, vitamin C 600 mg, vitamin E 100 mg, vitamin $K_3$ 104 mg, biotin 0.4 mg, folic acid 4 mg, calcium pantothenate 100 g, p-aminobenzoic acid 100 mg, niacin 120 mg, inositol 120 mg and choline chloride 4.0 g. Thus, the diets were formulated on the assumption that the rats would consume an equal number of calories, vitamins and minerals, but the rats receiving the high-fat diet also had a high-fibre diet. The food was stored in sealed plastic containers in the dark and maintained at 4°C.

### Purification of eicosapentaenoic acid (EPA) and docosahexaenoic acid (DHA)

Purified EPA ethyl ester (purity 99%) and DHA ethyl ester (purity 98%) were prepared as follows: commercial sardine oil (Nippon Suisan Co., Ltd, Tokyo, Japan) containing 18% of EPA and 12% of DHA was transethylated in ethanol with sodium ethoxide. An ethyl ester mixture was introduced into a distillation system equipped with a column packed with stainless-steel mesh, and a $C_{20}$ fatty acid ethyl ester-rich distillate and a $C_{22}$ fatty acid ethyl ester-rich distillate were fractionated. EPA ethyl ester and DHA ethyl ester were separated from the $C_{20}$ distillate and $C_{22}$ distillate, respectively, on an octadecyl silicate column, with methanol used as the eluting solvent. All solutions contained 0.05% *dl*-tocopherol as an antioxidant.

### Body weight and tumour measurement

Body weights, tumour incidence and measurements were recorded weekly throughout the experimental period. Each tumour location was recorded, and the size was measured with a Vernier caliper in two perpendicular dimensions. Tumour diameter was calculated by averaging these two measurements. Weekly tumour measurements were added for each rat, and the values were expressed by summing the average diameter of all tumours for tumour-bearing rats of each group and by summing the average diameter of initial tumours only (first palpable tumours) for tumour-bearing rats of each group. Mammary tumorigenesis was also assessed as the average tumour number and percentage tumour incidence. Estimated tumour weight (ETW) of the initial tumour was calculated by the following equation (Gren et al, 1972): ETW = largest diameter x shortest diameter$^2$/2 mg. Tumour doubling time ($Td_1$) was calculated as follows (Collins et al, 1956): $Td_1 = t\ln 1/2(\ln V_1 - \ln V_0)$, where $t$ is the time period between tumour appearance and 20 weeks after DMBA administration; $V_1$ is ETW 20 weeks following DMBA administration; $V_0$ is ETW at the time of tumour appearance.

### Tumour and serum fatty acids

For fatty acid analysis, lipids were first extracted from mammary tumours (30–250 mg) or serum (1 ml) with chloroform–methanol by the method of Folch et al (1957). To isolate the phospholipids

© Cancer Research Campaign 1997

Table 2 Principal fatty acids in tumour cell phospholipid fractions expressed as percentage of the total assayed fatty acid content (means ± s.d.)

| Groups | (a)CO-HF (n=6) | (b)CO-LF (n=5) | (c)EPA-HF (n=5) | (d)DHA-HF (n=5) | Fisher PLSD $P<0.05$ ($P<0.1$) |
|---|---|---|---|---|---|
| C14:0 | 0.8 ± 0.4 | 0.7 ± 0.1 | 0.5 ± 0.1 | 0.7 ± 0.1 | NS |
| C16:0 | 22.5 ± 1.1 | 22.4 ± 2.6 | 23.4 ± 1.9 | 24.0 ± 1.6 | NS |
| C16:1 | 1.5 ± 0.3 | 2.8 ± 0.3 | 1.4 ± 0.2 | 1.5 ± 0.1 | a–b, b–c, b–d |
| C18:0 | 15.0 ± 1.4 | 12.9 ± 1.0 | 15.7 ± 1.7 | 14.1 ± 1.0 | b–d |
| C18:1 (OL) | 15.9 ± 1.4 | 21.7 ± 1.8 | 16.2 ± 1.0 | 16.3 ± 1.2 | a–b, b–c, b–d |
| C18:2 (LA) | 4.6 ± 0.6 | 1.5 ± 0.5 | 5.5 ± 0.7 | 4.7 ± 0.9 | a–b, b–c, b–d |
| C20:4 (AA) | 25.8 ± 2.7 | 25.2 ± 1.1 | 24.4 ± 1.3 | 25.6 ± 2.7 | NS |
| C20:5 (EPA) | 0.0 ± 0.1 | 0.1 ± 0.1 | 0.9 ± 0.4 | 0.1 ± 0.1 | a–c, b–c, c–d |
| C22:4 | 3.5 ± 1.4 | 2.9 ± 0.3 | 2.5 ± 0.3 | 2.8 ± 0.5 | NS |
| C22:5 (DPA) | 0.2 ± 0.0 | 0.3 ± 0.2 | 1.9 ± 0.4 | 0.4 ± 0.1 | a–c, b–c, c–d |
| C22:6 (DHA) | 1.8 ± 0.2 | 1.5 ± 0.4 | 2.1 ± 0.1 | 3.9 ± 0.5 | a–d, b–d, c–d, (b–c) |

CO-HF, coconut oil-treated high-fat group; CO-LF, coconut oil-treated low-fat group; EPA-HF, eicosapentaenoic acid-treated high-fat group; DHA-HF, docosahexaenoic acid-treated high-fat group; OL, oleic acid; LA, linoleic acid; EPA, eicosapentaenoic acid; DPA, docosapentaenoic acid; DHA, docosahexaenoic acid; AA, arachidonic acid; NS, not significant.

from the neutral lipids, the extracted lipids were exposed to a thin-layer plate of silica gel G and developed with an *n*-hexane-diethylester-acetic acid (50:50:1, v/v/v) solvent system. The phospholipid band at origin was scraped off and phospholipids were recovered upon extraction with chloroform–methanol (2:1, v/v). After the removal of solvent under nitrogen, the phospholipids were transesterified with boron trifluoride-methanol and the methylesters were analysed by gas chromatography (Hewlett-Packard 5890A, Hewlett-Packard Co., Palo Alto, CA, USA) using a fused silica capillary column (Omegawax 320, Supelco, Bellefonte, PA, USA) (0.32 mm in diameter and 30 m in length) coated with 0.25-µm-thick Supelcowax 10 (Supelco, Bellefonte, PA, USA). The column temperature was maintained 185°C for 10 min, then raised to 225°C at a rate of 2°C $min^{-1}$ and maintained at the final temperature for 5 min. An injection temperature of 250°C, a detector temperature of 270°C and a column flow of 1.2 ml $min^{-1}$ of helium were used. Peaks were determined by a flame ionization detector and were qualified using a Hewlett Packard 3393A integrator (Hewlett Packard). Identification of each peak was carried out by the comparison of retention time with authentic fatty acid methyl esters. The compositions of individual fatty acids were expressed as the percentage of the total area of all fatty acid peaks from 14:0 to 22:6.

### Statistical analysis

Statistical differences in mammary tumour size, average tumour number, tumour doubling time, serum and tumour fatty acid composition were analysed using the Scheffé-type multiple comparison test. Values of $P < 0.05$ were considered significant.

### RESULTS

### Food intake, body weight and tumour histology

The average amount of feed per rat was 70 g per week. Consequently, the proportion of intake of *n*-3/*n*-6 PUFAs in a week was 1:6.36 in the EPA-HF and DHA-HF groups. The intake of *n*-3 PUFAs was minimal in the CO-HF and CO-LF groups. Total body weight did not differ significantly among the groups of rats throughout the experimental period. Twenty weeks after DMBA administration, the mean total body weight was 261 ± 29 g in the CO-HF group, 266 ± 30 g in the CO-LF group, 261 ± 23 g in the EPA-HF group and 261 ± 29 g in the DHA-HF group (Table 1). The induced mammary tumours were histologically identified as adenocarcinoma. No fibroadenomas were found.

### Tumour incidence and growth

Tumour incidence in the CO-HF group, CO-LF group, EPA-HF group and DHA-HF group was 80%, 39%, 42% and 25% respectively (Table 1). The total number of tumours per rat was about threefold higher in the CO-HF group than in the CO-LF group. The addition of EPA or DHA to the high-fat diet resulted in a reduction in the total number of tumours by one-third or one-sixth respectively. The number of tumours per rat as well as per tumour-bearing rat was significantly increased in the CO-HF group compared with the CO-LF, EPA-HF and DHA-HF groups (Table 1). Although the average estimated tumour weight of all tumours as well as the average estimated tumour weight of the first tumour of each rat was increased in the CO-HF group, this difference was not statistically significant. Moreover, the tumour doubling time was not statistically different among the groups (Table 1).

### Tumour phospholipid fatty acid composition

The administration of EPA and DHA resulted in increased EPA (20:5) and DHA (22:6) respectively. Oleic acid (OL, 18:1) was significantly decreased in the CO-HF, EPA-HF and DHA-HF groups compared with the CO-LF group. Linoleic acid (LA, 18:2) was significantly increased in the CO-HF, EPA-HF and DHA-HF groups compared with the CO-LF group. However, arachidonic acid (AA, 20:4) was not statistically different among the groups Table 2).

### Serum phospholipid fatty acid composition

The administration of EPA and DHA resulted in increased EPA (20:5) and DHA (22:6) respectively. OL (18:1) was significantly decreased in the CO-HF, EPA-HF and DHA-HF groups compared with the CO-LF group. LA (18:2) was significantly increased in the EPA-HF and DHA-HF groups compared with the CO-LF group. AA (20:4) was significantly decreased in the EPA-HF group compared with the CO-HF group ($P = 0.011$), while it was

 © Cancer Research Campaign 1997

**Table 3** Principal fatty acids in serum phospholipid fractions expressed as percentage of the total assayed fatty acid content (means ± s.d.)

| Groups | (a)CO-HF (*n*=6) | (b)CO-LF (*n*=5) | (c)EPA-HF (*n*=5) | (d)DHA-HF (*n*=5) | Fisher PLSD *P* <0.05 (*P* < 0.1) |
|---|---|---|---|---|---|
| C14:0 | 0.6 ± 0.2 | 0.6 ± 0.2 | 0.6 ± 0.2 | 0.5 ± 0.2 | NS |
| C16:0 | 10.5 ± 1.2 | 13.3 ± 1.7 | 12.0 ± 1.5 | 11.5 ± 0.9 | a–b |
| C16:1 | 0.9 ± 0.4 | 2.2 ± 0.9 | 0.8 ± 0.2 | 0.9 ± 0.6 | a–b, b–c, b–d |
| C18:0 | 28.4 ± 2.1 | 26.6 ± 2.6 | 25.6 ± 1.9 | 26.1 ± 2.3 | NS |
| C18:1 (OL) | 4.5 ± 0.6 | 12.8 ± 2.0 | 5.9 ± 0.4 | 6.3 ± 2.3 | a–b, b–c, b–d |
| C18:2 (LA) | 9.9 ± 4.1 | 4.7 ± 1.0 | 14.2 ± 1.9 | 12.9 ± 4.6 | b–c, b–d |
| C20:4 (AA) | 26.4 ± 2.2 | 23.5 ± 3.2 | 19.3 ± 3.2 | 21.4 ± 3.4 | a–c, (a–d) |
| C20:5 (EPA) | 0.1 ± 0.3 | 0.4 ± 0.3 | 3.7 ± 2.5 | 1.8 ± 2.1 | a–c, b–c |
| C22:4 | 4.5 ± 1.4 | 3.0 ± 1.0 | 1.3 ± 0.7 | 0.8 ± 0.5 | a–c, a–d, b–d, (b–c) |
| C22:5 (DPA) | 0.1 ± 0.2 | 0.2 ± 0.1 | 2.9 ± 0.5 | 0.6 ± 0.3 | a–c, b–c, c–d, (a–d) |
| C22:6 (DHA) | 5.5 ± 1.6 | 3.4 ± 0.3 | 5.8 ± 0.7 | 11.2 ± 1.8 | a–d, b–d, c–d, (b–c) |

CO-HF, coconut oil-treated high-fat group; CO-LF, coconut oil-treated low-fat group; EPA-HF, eicosapentaenoic acid-treated high-fat group; DHA-HF, docosahexaenoic acid-treated high-fat group; OL, oleic acid; LA, linoleic acid; EPA, eicosapentaenoic acid; DPA, docosapentaenoic acid; DHA, docosahexaenoic acid; AA, arachidonic acid; NS, not significant.

**Table 4** Levels of linoleic acid and arachidonic acid, and ratio of arachidonic acid/linoleic acid in tumour and serum (means ± s.d.)

| Groups | (a)CO-HF (*n*=4 | (b)CO-LF (*n*=5) | (c)EPA-HF (*n*=4) | (d)DHA-HF (*n*=4) | Fisher PLSD *P* <0.05 |
|---|---|---|---|---|---|
| Ratio of tumour LA / serum LA | 0.6 ± 0.1 | 0.3 ± 0.1 | 0.4 ± 0.1 | 0.4 ± 0.2 | NS |
| Ratio of tumour AA / serum AA | 1.0 ± 0.2 | 1.1 ± 0.2 | 1.3 ± 0.3 | 1.3 ± 0.2 | NS |
| Ratio of AA / LA in tumour | 5.7 ± 1.2 | 19.1 ± 8.6 | 4.5 ± 0.8 | 5.6 ± 1.3 | a–b, b–c, b–d |
| Ratio of AA / LA in serum | 2.9 ± 0.9 | 5.2 ± 1.5 | 1.4 ± 0.3 | 1.9 ± 0.8 | a–b, b–c, b–d |

CO-HF, coconut oil-treated high-fat group; CO-LF, coconut oil-treated low-fat group; EPA-HF, eicosapentaenoic acid-treated high-fat group; DHA-HF, docosahexaenoic acid-treated high-fat group; LA, linoleic acid; AA, arachidonic acid; NS, not significant.

insignificantly decreased in the DHA-HF group compared with the CO-HF group ($P = 0.098$) (Table 3).

### Comparison of linoleic acid and arachidonic acid in tumours and serum

LA levels in serum were consistently higher than tumour levels in each group. LA levels in tumour and serum were significantly higher in the CO-HF, EPA-HF and DHA-HF groups compared with the CO-LF group. The tumour–serum ratio of LA was not significantly different among the groups (Table 4). Although tumour AA levels were not different among the groups, AA levels in serum were decreased in the EPA-HF and the DHA-HF groups compared with the CO-HF group. Similarly, the tumour–serum ratio of AA was higher in the DHA-HF and EPA-HF groups than in the CO-HF and CO-LF groups, although the difference was not statistically significant (Table 4). The ratio of AA–LA in tumour and serum was not different among the CO-HF, EPA-HF and DHA groups, while it was significantly higher in the CO-LF group compared with the CO-HF, EPA-HF and DHA groups (Table 4).

### DISCUSSION

The promotion of carcinogen-induced, spontaneous and transplantable mammary tumours is enhanced in rats fed increasing concentrations of *n*-6 PUFAs containing a large amount of linoleic acid (LA) (Carter et al, 1983; Welsch, 1987; Noguchi et al, 1991), although cohort and case–control studies have generally been unsuccessful in confirming a strong association between dietary fat and human breast cancer risk (Goodwin et al, 1987). Alternatively, diets containing high levels of *n*-3 PUFAs, mainly EPA and DHA, have been shown to inhibit development of several carcinogen-induced and transplantable cancers (Karmali et al, 1984; Carroll and Braden, 1985*a*; Jurkowski and Cave, 1985; Cohen et al, 1993; Kinoshita et al, 1994; Rose et al, 1995). With regard to the promotive or inhibitory effects of PUFAs, several authors have drawn attention to the dietary imbalance of *n*-3 and *n*-6 PUFAs (Karmali, 1987*a*; Karmali et al, 1989; Kromhout, 1990). In experimental studies, it has been reported that the ratio of *n*-3 to *n*-6 PUFAs, rather than the total quantity of *n*-3 PUFAs, plays an important role in the inhibition of tumorigenesis by *n*-3 PUFAs (Abou-El-Ela et al, 1989; Cohen et al, 1993; Noguchi et al, 1995*a*). In an in vivo study, Abou-El-Ela et al (1989) have found that a diet containing an *n*-3/*n*-6 PUFA ratio of 1:2 reduced DMBA-induced mammary tumorigenesis. Moreover, in other in vivo studies, lower levels of dietary *n*-3 fatty acids either had no effect or enhanced mammary tumour development (Carroll and Braden, 1985*b*; Jurkowski and Cave, 1985; Cohen et al, 1993). Also, in an in vitro study (Noguchi et al, 1995*a*), cell proliferation of human breast cancer cells (MDA-MB-231) was inhibited at a DHA–LA ratio of 1:2.08 and at an EPA–LA ratio of 1:0.69, while a lower ratio of *n*-3/*n*-6 PUFAs did not enhance cell proliferation. Thus, *n*-3 PUFAs have unique properties and a potential role as chemopreventive agents in breast cancer, but a large amount of dietary *n*-3 PUFAs would be required for breast cancer prevention and treatment.

It has been reported that Greenland Eskimos (Sinclair, 1981), who exhibit low breast cancer rates, consume PUFAs with *n*-3/*n*-6 ratios of 1:0.36 compared with high-risk Americans, who consume PUFAs with an *n*-3/*n*-6 ratio of approximately 1:8.33 (Sinclair,

© Cancer Research Campaign 1997 

1981; Cohen et al, 1993). The Eskimo diet is clearly unusual in that fat is derived almost exclusively from fish and aquatic mammals and is therefore rich in *n*-3 PUFAs. However, an epidemiological study has demonstrated that the Japanese, who also exhibit low breast cancer rates, consumed PUFAs with *n*-3/*n*-6 ratios ranging from 1:4.26 to 1:4.20 between 1971 and 1980 (Hirahara, 1995). It is apparent that an *n*-3/*n*-6 PUFA ratio of less than 1:2 is still effective in reducing the incidence of breast cancer. In the present study, EPA and DHA were given at the *n*-3/*n*-6 PUFA ratio of 1:6.36. Consequently, tumour incidence was decreased by the administration of low-dose EPA and DHA, while their inhibitory effects on tumour growth did not reach significance. There is an apparent discrepancy between the results of the present study and previous experimental studies (Carroll and Braden, 1985*b*; Jurkowski and Cave, 1985; Cohen et al, 1993). While *n*-3 PUFAs were provided in food in the previous in vivo studies, they were given twice weekly as a drug with an *n*-3/*n*-6 PUFA ratio of 1:1.82 in the present study. It would be of interest to investigate how such effects relate to observed changes in the chemical content of fatty acids in tumour and serum.

Several studies have been undertaken to investigate the influence of *n*-3 PUFAs on the composition of fatty acids in tumours (Karmali et al, 1984, 1989; Jurkowski and Cave, 1985; Takata et al, 1990; Rose et al, 1995) and in plasma or serum (Abou-El-Ela et al, 1988; Cohen et al, 1993). They have demonstrated a decrease in AA in tumours and serum from animals fed fish oil, a source rich in EPA and DHA. Both EPA and DHA may exert their effects by competing with arachidonic acid (AA), thereby diminishing the formation of AA metabolites (Karmali, 1987*a,b*). It has been reported that fish oil lowers the activity of $\Delta 5$ and $\Delta 6$ desaturases in rodents (Hagve and Christophersen, 1984; Juan and Sametz, 1985; Garg et al, 1988). Takata et al (1990) have suggested that LA is increased and AA is decreased by the inhibition of $\Delta 5$ and $\Delta 6$ desaturases. Karmali et al (1984) have demonstrated that the ratio of AA to LA may be an important factor for mammary tumorigenesis. In the present study, LA levels in tumour and serum were increased by the intake of a HF diet, but not by the administration of low-dose EPA and DHA. AA levels in tumour and serum were not altered by a HF diet, although serum, but not tumour, AA level was decreased by the administration of low-dose EPA and tended to be decreased by the administration of low-dose DHA. It is likely that AA metabolism in serum plays an important role in mammary tumorigenesis, although the ratio of AA to LA in tumour and serum was not associated with tumour incidence. An epidemiological study has demonstrated that serum AA was decreased and serum LA was increased in Japanese compared with Americans (Iso et al, 1989).

AA-derived eicosanoids are believed to play an important role in these processes of tumorigenesis and tumour proliferation (Carter et al, 1983; Noguchi et al, 1995*c*). However, it is important to distinguish the mechanisms that underly tumorigenesis and tumour proliferation (Noguchi et al, 1991, 1995*b*). In the present study, DHA was more active than EPA in reducing tumour incidence, although EPA was more active than DHA in reducing serum AA levels, findings compatible with the study of Terano et al (1987). Although EPA is a competitive inhibitor of both the cyclo-oxygenase and lipoxygenase pathways (Karmali, 1987*b*), DHA is a strong inhibitor of prostaglandin synthesis (Corey et al, 1983). Nevertheless, DHA can be retroconverted to EPA in vivo (Terano et al, 1987) and in vitro (de Antueno et al, 1989). In in vitro studies, it has been reported that DHA is more active than EPA in reducing PGE secretion (Noguchi et al, 1995*a*; Rose et al, 1995), while EPA is more active than DHA in reducing LTB secretion (Noguchi et al, 1995*a*). Prostaglandins play a role in the regulation of both humoral and cell-mediated immunity (Goldyne and Stobo, 1981). It has been shown that prostaglandins exert an inhibitory effect on natural killer cells, components of the host defence system, which are thought to play a role in immunosurveillance (Brunda et al, 1980). Tumorigenesis has been suggested as being inhibited by the decreased production of immunosuppressive prostaglandins (Carter et al, 1983; McCormick et al, 1985; Noguchi et al, 1991). Therefore, it appears that DHA is more active than EPA in inhibiting mammary tumorigenesis by interfering with prostaglandin metabolism.

Low-dose EPA and DHA completely blocked the stimulatory effect of fat on tumour incidence in the present study, while their inhibitory effects on tumour growth did not reach significance. Therefore, relatively low-dose EPA and DHA may be useful for breast cancer prevention. However, this study was performed in an experimental carcinogenesis model. Ip (1993) has stated that fat promotes mammary carcinogenesis only under a very stringent set of conditions, which might not be duplicated in the arena of fat intake and human breast cancer risk. Further analysis to investigate the mechanisms of the inhibitory effects of EPA and DHA would considerably strengthen this work.

### ACKNOWLEDGEMENT

This work was supported by Grants-in-Aid for Cancer Research from the Ministry of Education, Science and Culture of Japan.

### REFERENCES

Abou-El-Ela SH, Prasse KW, Carroll R, Wade AE, Dharwadkar S and Bunce OR (1988) Eicosanoid synthesis in 7,12-dimethyl-benz(a)anthracene-induced mammary carcinomas in Sprague–Dawley rats fed primrose oil, menhaden oil or corn oil diet. *Lipids* **23**: 948–954

Abou-El-Ela SH, Prasse KW, Farrell RL, Carroll RW, Wade AE and Bunce OR (1989) Effects of D,L-2-difluoromethylornithine and indomethacin on mammary tumor promotion in rats fed high n-3 and/or n-6 fat diets. *Cancer Res* **49**: 1434–1440

Brunda MJ, Herberman RB and Holden HT (1980) Inhibition of murine natural killer cell activity by prostaglandins. *J Immunol* **124**: 2682–2687

Caroll KK and Braden UN (1985*a*) Dietary polyunsaturated fat in relation to mammary carcinogenesis. *J Am Oil Chem Soc* **62**: 640

Carroll KK and Braden LN (1985*b*) Dietary fat and mammary carcinogenesis. *Nutr Cancer* **6**: 254–259

Carter CA, Milholland RJ, Shea W and Ip MM (1983) Effect of the prostaglandin synthetase inhibitor indomethacin on 7,12-dimethylbenz(a)-anthracene-induced mammary tumorigenesis in rats fed different levels of fat. *Cancer Res* **38**: 3559–3562

Cohen LA, Chen-Backlund J-Y, Sepkovic DW and Sugie S (1993) Effect of varying proportions of dietary menhaden and corn oil on experimental rat mammary tumor proliferation. *Lipids* **28**: 449–456

Collins VP, Loeffler RK and Tivey H (1956) Observations on growth rates of human tumours. *Am J Roentgenol* **76**: 988–1000

Corey RJ, Shih C and Cashman JR (1983) Docosahexaenoic acid is a strong inhibitor of prostaglandin but not leukotriene biosynthesis. *Proc Natl Acad Sci USA* **80**: 3581–3584

De Antueno RJ, De Bravo MG, Toledo J, Mercuri O and De Thomas ME (1989) *In vitro* effect of eicosapentaenoic and docosahexaenoic acids on prostaglandin $E_2$ synthesis on a human lung carcinoma. *Biochem Int* **19**: 489–496

Early Breast Cancer Trialists' Collaborative Group (1992). Systemic treatment of early breast cancer by hormonal, cytotoxic, or immune therapy. *Lancet* **339**: 1–15

Folch J, Lees M and Sloane Stanley GH (1957) A simple method for the isolation and purification of total lipids from animal tissues. *J Biol Chem* **226**: 497–509

© Cancer Research Campaign 1997

Garg ML, Sebokova E, Thompson ABR and Clandinin MT (1988) Δ6-Desaturase activity in liver microsomes of rats fed diets enriched with cholesterol and/or ω-3 fatty acids. *Biochem J* **249**: 351–356

Goldyne ME and Stobe JD (1981) Immunoregulatory role of prostaglandins and related lipids. *CRC Crit Rev Immunol* **2**: 189–224

Goodwin PJ and Boyd NF (1987) Critical appraisal of the evidence that dietary fat intake is related to breast cancer risk in humans. *J Natl Cancer Inst* **79**: 474–485

Gren RI, Greenberg NH and Macdonald MM (1972) Protocol for screening chemical agents and natural products against animal tumors and other biological systems. *Cancer Chemother Rep* **3**: 51–61

Hagve TA and Christophersen BO (1984) Effect of dietary fats on arachidonic and eicosapentaenoic acid biosynthesis and conversion to C22 fatty acids in isolated rat liver cells. *Biochem Biophys Acta* **796**: 205–217

Hirahara (1995) Annual changes of Japanese dietary fat intake: quantity and quality (in Japanese). *Shishitsu Eiyougaku* **4**: 73–82

Ip C (1993) Controversial issues of dietary fat and experimental mammary carcinogenesis. *Prev Med* **22**: 728–737

Iso H, Sato S, Folsom AR, Shimamoto T, Terao A, Munger RG, Kitamura A, Konishi M, Iida M and Komachi Y (1989) Serum fatty acids and fish intake in rural Japanese, urban Japanese, Japanese American and Caucasian American men. *Int J Epidemiol* **18**: 374–381

Juan H and Sametz W (1985) Dihomo-—linolenic acid increases the metabolism of eicosapentaenoic acid in perfused vascular tissue. *Prostaglandins Leuko Med* **19**: 79–86

Jurkowski JJ and Cave WT Jr (1985) Dietary effects of menhaden oil on the growth and membrane lipid composition of rat mammary tumours. *J Natl Cancer Inst* **74**: 1145–1150

Kaizer F, Boyd NF, Kriukow V and Trichler D (1989) Fish consumption and breast cancer risk: an ecological study. *Nutr Cancer* **12**: 61–68

Karmali RA, Marsh J and Fuchs C (1984) Effects of omega-3 fatty acids on growth of a rat mammary tumor. *J Natl Cancer Inst* **73**: 461–475

Karmali RA (1987*a*) Eicosanoid in neoplasia. *Prev Med* **16**: 493–502

Karmali RA (1987*b*) Fatty acids: Inhibition. *Am J Clin Nutr* **45**: 225–229

Karmali RA, Donner A, Gobel S and Shimamura T (1989) Effects of n-3 and n-6 fatty acids on 7,12-dimethylbenz(a)anthracene-induced mammary tumorigenesis. *Anticancer Res* **9**: 1161–1168

Kinoshita K, Noguchi M, Earashi M, Tanaka M and Sasaki T (1994) Inhibitory effects of purified eicosapentaenoic acid and docosahexaenoic acid on growth and metastasis of murine transplantable mammary tumor. *Int J In Vivo Res* **8**: 371–374

Kromhout D (1990) The importance of n-6 and n-3 fatty acids in carcinogenesis. *Med Oncol Tumor Pharmacother* **7**: 173–176

McCormick DL, Madigan MJ and Moon RC (1985) Modulation of rat mammary carcinogenesis by indomethacin. *Cancer Res* **45**: 1803–1808

Minami M, Noguchi M (1996) Effects of low-dose eicosapentaenoic acid (EPA), docosahexaenoic acid (DHA) and dietary fat on the incidence, growth and cell kinetics of mammary carcinoma in rats. *Oncology* **53:** 398–405

Noguchi M, Taniya T, Koyasaki N, Kumaki T, Miyazaki I and Mizukami Y (1991) Effects of the prostaglandin synthetase inhibitor indomethacin on tumorigenesis, tumor proliferation, cell kinetics, and receptor contents of 7,12-dimethylbenz(a)anthracene-induced mammary carcinoma in Sprague–Dawley rats fed a high- or low-fat diet. *Cancer Res* **51**: 2683–2689

Noguchi M, Earashi M, Minami M, Kinoshita K and Miyazaki I (1995*a*) Effects of eicosapentaenoic and docosahexaenoic acid on cell growth and prostaglandin E and leukotriene B production by a human breast cancer cell line (MDA-MB-231). *Oncology* **52**: 458–464

Noguchi M, Earashi M, Miyazaki I, Tanaka M and Sasaki T (1995*b*) Effects of indomethacin with or without linoleic acid on human breast cancer cells *in vitro*. *Prostaglandins Leukot Essent Fatty Acids* **52**:381–386

Noguchi M, Rose DP, Earashi M and Miyazaki I (1995*c*) The role of fatty acids and eicosanoid synthesis inhibitors in breast carcinoma. *Oncology* **42**: 265–271

Rose DP, Connolly JM, Rayburn J and Coleman M (1995) Influence of diets containing eicosapentaenoic or docosahexaenoic acid on growth and metastasis of breast cancer cells in nude mice. *J Natl Cancer Inst* **87**: 587–592

Sinclair HM (1981) The relative importance of essential fatty acids of the linoleic and linolenic families: studies with an Eskimo diet. *Prog Lipid Res* **20**: 897–899

Takata T, Minoura T, Takada H, Sakaguchi M, Yamamura M, Hioki K and Yamamoto M (1990) Specific inhibitory effect of dietary eicosapentaenoic acid on *N*-nitroso-*N*-methylurea-induced mammary carcinogenesis in female Sprague–Dawley rats. *Carcinogenesis* **11**: 2015–2019

Terano T, Hirai A, Tamura Y, Kumagai A and Yoshida S (1987) Effect of dietary supplementation of highly purified eicosapentaenoic acid and docosahexaenoic acid on arachidonic acid metabolism in leukocytes and leukocyte function in healthy volunteers. In *Advances in Prostaglandin, Thromboxane and Leukotriene Research*, Vol. 17. Samuelsson B, Paoletti R and Ramwell PW (eds), pp. 880–885. Raven Press: New York.

Tominaga S and Kuroishi T (1995) Epidemiology of breast cancer in Japan. *Breast Cancer* **2**: 1–7

Welsch CW (1987) Enhancement of mammary tumorigenesis by dietary fat: review of potential mechanisms. *Am J Clin Nutr* **45**: 192–202

© Cancer Research Campaign 1997